# A Surgical Operation in the Seventeenth Century

**Published:** 1906-01-06

**Authors:** 


### A Surgical Operation in the Seventeenth Century.

It is not easy to obtain details of the mode of operating upon patients in the seventeenth century, and the following account of an amputation of the breast will be read with interest. It is written by the Rev. John Ward, Vicar of Stratford-on-Avon from 1662 until 1681:—

"A cancer in Mrs. Townsend's breast, of Alverston, taken off by two surgeons; one's name was Clerk of Bridgnorth, another's name was Leach of Sturbridg. First they cutt the skin cross and laid itt back, then they worked their hands in ytt, one above and the other below, and so till their hands mett, and so brought itt out. They had their needles and waxt thread ready, but never used them; and allso their cauterising irons, but they used them not She lost not above ℥vi. of blood in all. Dr. [Walter] Needham coming too late, staied next day to see it opened. Hee said it was a melliceris, and not a perfect cancer, but itt would have been one quickly. There came out a gush of a great quantity of waterish substance, as much as would fill a flaggon; when they had done they cutt off, one one bitt, another another, and putt in a glass of wine and some lint, and so let itt alone till the next day: then they opend itt again and injected myrrhe, aloes and such things as resisted putrefaction and so bound itt upp againe.

"Every time they dresst itt, they cutt off something of the cancer that was left behind; the chyrurgions were for applying a caustick, but Dr. Needham said no, not till the last, since she could endure the knife. They prepared her bodie somewhat, and let her blood the day before, one of the chyrugeons told her afterwards that shee had endured soe much, that hee would have lost his life ere hee would have suffered the like; and the Dr. said he had read that women would endure more than men, but did not beleeve itt till now. The way how and where itt should be cutt was markt with ink by one Dr. Edwards who lives at Bridgnorth. Mrs. Townsend likt him very well: hee said iff they could prevent a gangrene there was little fear, iff she fell not into a feavour.

"1666. Msr. Townsend, of Alverston, being dead of a cancer, Mr. Eedes and I opened her breast in the outward part and found it very cancrous; itt had been broken and a mellicerous part was yet remaining when wee saw itt, which being launct, yielded two porringers full of a very yellow substance which came out plentifully out of the cavities of the breast. The flesh that was growne againe after part was taken out, was of a hard gristly substance, which seemed very strange. The ribs were not putrefied as wee could discerne, nor anything within the breast of a cancerous nature for we ranne the knife withinside the breast through the intercostal muscles. . . . The cancer was a strange one, as was evident: wee wanted spunges and other things convenient, or else wee had opened the cavitie of the breast."

There is little doubt that Dr. Needham was correct in his diagnosis that the tumour was not a perfect cancer, for it was in modern nomenclature a cystic sarcoma. Dr. Walter Needham was born in Surrey and was educated at Westminster and Trinity College, Cambridge. He practised for a time at Shrewsbury and was an ornament of the Oxford scientific school, though he died in poverty in London in 1691. Sydenham speaks of him as "tam medicae artis quam rei literariae decus et laus." Dr. Richard Edwards, who marked out the operation for the surgeons, was a practitioner at Bridgnorth, who was admitted an extra-licentiate of the College of Physicians on December 19, 1662. The Rev. John Ward, writer of the account given above, was the son of John Ward of Northants. He matriculated at Magdalen Hall in 1646, at the age of seventeen, and took the degree of M.A. from Christchurch in 1652. He was Vicar of Gretton in 1652 and of Weedon Beck, both in Northamptonshire, in 1663, but is said to have been deprived in 1674 "for divers crimes committed and duties omitted by him." He was appointed to the vicarage of Stratford-on-Avon in 1662 and was buried in his parish church on September 13, 1681. Throughout his life he was interested in the practice of medicine and after taking his degree at Oxford he lived for a time at the Bell in Aldersgate Street to be near the Barber Surgeons' Hall in Monkwell Street, where he attended the lectures of Dr. Scarborough and Dr. Terne. He seems afterwards to have taken out a bishop's licence to practise medicine. He is well known as the author of a Diary extending from 1648-1679 which, amongst other interesting details, gives some gossiping facts about Shakespeare.

